# Trust and Distrust in Pension Providers in Times of Decline and Reform: Analysis of Survey Data 2004–2021

**DOI:** 10.1007/s10645-022-09411-x

**Published:** 2022-09-26

**Authors:** Hendrik P. van Dalen, Kène Henkens

**Affiliations:** 1 Netherlands Interdisciplinary Demographic Institute (NIDI-KNAW)/University of Groningen, P.O. Box 11650, 2502 AR The Hague, The Netherlands; 2 Tilburg School of Economics and Management (TISEM), Tilburg University, P.O. Box 90153, 5000 LE Tilburg, The Netherlands; 3 University Medical Center Groningen (UMCG), University of Groningen, P.O. Box 72, 9700 AB Groningen, The Netherlands; 4 Department of Sociology, University of Amsterdam, Nieuwe Achtergracht 166, 1018 WV Amsterdam, The Netherlands

**Keywords:** Pensions, Trust, Funding ratio, Pension funds, Government, D14, G2, G4, H55

## Abstract

Trust in pension providers by participants is essential because pension providers try to fulfill their pension promises in a fundamentally uncertain world. Reforms and crises are therefore the ultimate testing ground for pension trust. In this paper we estimate with repeated cross-sectional survey data how trust and distrust in Dutch pension funds and the government have evolved over the period 2004–2021 and what the impact of financial stability on trust in these two institutions has been. Financial stability of pension funds, measured by their funding ratio, is shown to affect trust positively, but it does not decrease distrust significantly. Based on the estimation results, achieving a situation where the majority of the adult population trusts pension funds is likely to be attained at funding ratios of 115 or higher. Financial stability of government (measured by government debt/GDP ratio) does not affect either trust or distrust levels. Underlying drivers of distrust and trust such as personal characteristics are also notable: self-employed are more prone to distrust pension funds than employees. Women are more than men likely to take a neutral position.

## Introduction

Trust in pension providers lies at the heart of offering a pension insurance contract (Barr & Diamond, 2006). The future is uncertain and as a consequence of this property pensions offered by pension funds or the government are by their very nature incomplete contracts as not every possible contingency can be covered. The governance structure behind pension contracts is therefore essential in making pension plans credible and hence trustworthy (Admati, 2021; Besley & Prat, 2005). The fact that within the OECD (2017) most governments are considering or implementing pension reforms is a reflection of the so-called incompleteness of pension contracts. Pension programs may have turned out to be financially unsustainable in light of population ageing or not in tune with the requirements of the labour market where lifetime jobs have become rare and contractual flexibility a force to be reckoned with. Certainly pension providers offering defined benefit contracts are more likely to experience reforms as these promises are particularly vulnerable to increasing life expectancies or low interest rates (Bovenberg & Gradus, 2015). The pension system of the Netherlands may be a case in point. Even though the system is ranked among the best in the world among pension experts (see the annual Mercer Global Pension Index), according to the Dutch government the old system is “teetering”. And as they stress in their justification of pension reform plans: “Without innovation, the chances are high that trust in our pension system will erode even further” (p. 4, Ministry of Social Affairs & Employment, 2020).

The consequences of pension policy decisions in terms of trust of participants or the citizens at large are easily posed but rarely made concrete. In this paper we examine, first of all, the development of trust in pension providers over the past 2 decades for the case of the Netherlands and, secondly, raise the question whether changes in trust are linked to the financial sustainability of these pension providers.

Understanding the development of trust and distrust in pension providers is of utmost importance for two reasons. First of all, because shocks in pension policies are likely to be accompanied by losses of trust whenever vested interests of specific groups are at stake, but the real issue is whether trust can recover once the dust of a shock has settled, or whether trust has been replaced by distrust. Once the latter becomes a reality, pension providers may become more risk averse or more averse to take necessary corrective reforms. A second reason why the development of trust across time is an important topic is because changes in trust levels may affect individual decisions in savings, investment and work across the lifetime. So far, these dynamic issues of trust have not been well covered in the pension literature for plausible reasons. The current scholarly literature on pension trust is small but gaining depth over time and relies mostly on cross-sectional studies. The examination of trust in pension providers (Van Dalen & Henkens, 2018; Van der Cruijsen et al., 2021a; Vickerstaff et al., 2012) relies on a diverse body of literature on trust in organizations and institutions which in turn draws on insights of disciplines like economics, marketing, psychology, management and political science. The core of the matter in measuring and explaining trust revolves around the assumption that trust is both a trait of the trustee (namely a pension provider)—*perceived trustworthiness*—as well as the person or trustor who has to trust others—the *propensity to trust* [cf. Mayer et al. (1995)]. The perceived trustworthiness of financial institutions is shown to consist of a multitude of characteristics, although in most studies the elements of ability, benevolence and integrity are central to understanding trust (Pirson & Malhotra, 2011; Van Raaij, 2016; Vickerstaff et al., 2012). Van Dalen and Henkens (2018) show that the perceived integrity, competence, stability, and benevolence of pension providers matter in assessing the trustworthiness of pension providers. Much less is known about changes in trust over time and the objective factors affecting the pension industry that influence these changes.

The difficulty in assessing and comparing developments across time is that such an exercise is not only affected by the events of the day—such as crises or policy reforms—but also the composition and characteristics of birth cohorts that make up a population. Changes in trust might be related to the entry and exit of generations/cohorts entering the work force. These generations may be characterized by a different composition of the workforce (e.g., the percentage of self-employed) and different levels of education. And the cohorts, themselves may also contain a different spirit or attitude towards pensions as generations may have encountered different capital market experiences or economic crises (Malmendier & Nagel, 2011; Sunde & Dohmen, 2016) and hence be affected in their outlook or behaviour with respect to trusting financial institutions.

A key element in perceiving pension providers as trustworthy is the *perceived* financial stability (Van Dalen & Henkens, 2018). However, the trust literature rarely employs real-time indicators of pension providers like asset positions of pension funds or government debt levels in case of government in its role as pension provider. Of course, the lack of research on such issues is in part affected by the lack of longitudinal data to track changes in trust in pension funds as a group, or lack of data on individual pension funds in a cross-sectional setting.

A final point that is not yet covered in the current literature on pension trust is the distinction between trust and distrust. Despite the fact that trust surveys are based on items that could offer a more fine-grained analysis, the research itself generally focuses on trust as if it is a binary choice—you either trust someone or some institution or not. However, this base category—not expressing trust—could be a mixture of being neutral or distrustful. We deviate from this standard practice in this paper by examining whether there are asymmetric reactions across time between those who trust and who distrust pension providers to developments in financial stability. An important reason for looking into this issue is based on insights in other disciplines where trust and distrust leads to more depth in understanding reactions of people to actions of organizations (Kramer, 1999; Van de Walle & Six, 2014). And in our study of pension trust this distinction might enlarge our insight into the building and regaining of trust. A common saying that captures the concerns of organizations is that “trust takes years to build, seconds to break and forever to repair” and the asymmetry that is part of radical changes in trust can only be examined by looking closely how people who have lost trust and those who still have trust differ in their response to, e.g., the financial stability of pension providers.

In this paper we will explain differences in trust and distrust in Dutch pension providers across time using data gathered at eight measurement points covering the period 2004–2021 by using repeated cross-sectional survey data. The central research questions in this paper are: (1) whether there are substantial differences across time in trust and distrust and (2) does the financial stability of pension funds and government—as approximated by their funding ratio and government debt position—play a role and if so to what extent? The data to be used to answer these questions relies on data collected in a uniform manner by one research institution (Centerdata, Tilburg University). The trust of citizens in pension providers is measured and analysed for two pension providers: the government as provider of a public pension and privately organized pension funds which offer a supplementary pension on top of the public pension.

The outline of the paper is as follows. In the next section we will give a short literature overview of the relationship between pension trust and financial stability and subsequently offer some context in Sect. 3 on the Dutch situation on both these aspects. Section 4 will cover issues concerning the operationalization of concepts used and the details of the data used and the methodology used to answer the two research questions. Section 5 reports on the estimation results and Sect. 6 concludes with a discussion of the results obtained.

## Theories of Trust and Distrust

The importance of trust in economic life resounds in a statement by Arrow (1972): “Virtually every commercial transaction has within itself an element of trust, certainly any transaction conducted over a period of time.” A common definition of trust is that an individual or an institution—the trustee—will perform actions that are beneficial (or at least not detrimental) to the party—the trustor—that enters into a contract. This contract can be a formal contract but often the contract is an informal one, i.e., behavioural rules that are embodied in social norms and practices. In the case of pension contracts, the element of time is an important element as pension finance covers a lifetime and, depending on the type of contract, this may also involve substantial risk pooling within and between generations. Trust in the financial institutions that organize and finance pension programs on behalf of citizens is therefore essential, but lapses in trust are also understandable given the nature of an uncertain world and the number of stakeholders involved.

In economics much weight is attached to analysing trust by focusing on direct interactions and subsequently distilling ‘revealed’ levels of trust based on laboratory experiments.^1^ Increasingly, attitudinal measures of trust are considered informative because these measures offer more opportunities to include real life elements that come into play in economic transactions [cf. Sapienza et al. (2013)]. Certainly, in the case of pension institutions laboratory experimental outcomes have limited ecological validity because in many countries where mandatory enrolment in pension programs exists direct interactions—that figure so prominently in lab experiments—between trustors (participants in pension programs) and the trustee (the pension providers) are rare.

To gain insights in the development of trust in pension institutions domain specific measures of trust in institutions are important. In this paper we focus our attention on so-called broad-scope trust which is the trust in a group of financial institutions, such as pension funds. However, we also focus on the government as pension provider and one can say that this paper also focuses on narrow-scope trust because there is only one provider when it comes to public pensions, viz. the government. The key hypothesis in this study is that financial stability is a direct driver of trust and distrust in pension funds and the governments as pension provider.

The reason why (financial) stability is deemed such an important element in increasing the trustworthiness of financial institutions is that organizations that are solvent can make good on their promises and offer a stable pension benefit or stable pension premiums in the case of pension funds. As shown in Van Dalen and Henkens (2018), stability as perceived by the general population is one of the most important predictors of trust in pension funds. This conclusion is based on a cross-sectional study which raises questions about causality and other biases such a common method bias. For a more solid test of the relationship between financial stability and trust we need to incorporate actual financial indicators that are specific to the various pension providers in the analyses.

The reason for looking at both *trust* and *distrust* of pension providers is linked to developments within the trust literature in organization science (Kramer, 1999), psychology (Schul et al., 2008) and political science (Bertsou, 2019; Van de Walle & Six, 2014). This literature indicates that a distinction between trust and distrust may be important as both groups may consist of distinctly different types of people and these differences may translate in divergent reactions. E.g., Schul et al. (2004, 2008) show by means of experiments that individuals use different strategies depending on whether the environment is characterized by trust of distrust (as manipulated by the setup of the experiment). When individuals sense they should be on guard they are likely to avoid routine strategies, routines that have proven to be optimal and regularly used in normal environments. When an environment is in state of flux it might be beneficial to be distrustful as the routines and decisions you would have made in normal times are no longer optimal. However, sticking to being distrustful in normal times may also have the side-effect of using routines that are not adapted to such times. The political science literature shows a growing distrust towards political and public institutions (Cook & Gronke, 2005; Van de Walle & Six, 2014), which in turn seems to reveal deep-seated discontent and eroding support for the government. However, one should be careful not to take a one-sided view of the concept of distrust as it does not necessarily have a negative connotation as one can distil from the work of classical liberal writers [see Hardin (2002) for an overview]. Distrust can be an essential building block of the checks and balances in democracies as vigilant citizens might offer a stimulus for trustees to perform well and perhaps also offer insights or information that would not come to light by those who completely trust a government. Fein (1996) also points out the other possibility that information that makes people suspicious might lead to a state of attributional conservatism, in other words, thresholds for accepting behavioural information is elevated. Once distrust is based on deep-seated discontent and interaction and information is cut-off, the virtues of distrust disappear and distrust become a threat to the existence of an institution in a manner akin to the analysis of exit, voice and loyalty within organizations and states of Hirschman (1970). The positive side of distrust can been found in the option of voice: airing complaints and trying to get heard in the hope that things will improve while remaining loyal. In the case mandatory pension systems like the Dutch system, the option of voice is particulary important because the exit option—or to vote with one’s feet—has been ruled out by definition. The negative sides of distrust becomes visible when loyalty and hope for improvement are lost and voice is no longer seen as an effective option. At that point the option of exit becomes real, but it is difficult to predict how this willl materialize in a setting where participation is mandatory. Given that private pensions in most societies are intertwined with decisions made and regulated by governments it is not only important to focus on pension funds but also the government, and see how distrust and trust in both these institutions fare over time.

## Context: Pensions in the Netherlands

In order to understand the issue of trust in the Dutch context it is necessary to keep in mind the key players that figure prominently in the Netherlands in the provision of pensions, and the timeline of the most prominent developments that have taken place in the recent decades that may have entered the minds of citizens.

### Benefits and Premiums

In the Netherlands, most employees save and accumulate pension rights within a three-pillar system: (1) a basic public pension plan (the so-called ‘AOW’) provided by the government; (2) a mandatory supplementary pension plan sponsored to large extent by employers and provided and managed by pension funds; and (3) individual voluntary pension savings. Up and till this day the public pension—financed on a pay-as-you-go basis—in combination with the supplementary pension provisions are for most Dutch citizens the basic elements of what citizens consider “their pension”. It should, however, be mentioned that currently developments are taking place which may give a different outlook for the future workers. Increasingly self-employed may be tempted or forced to accumulate pension savings (Hershey et al., 2017) and high-income employees may also be forced to supplement their pension savings by voluntary savings as a cap is placed on the level of income covered by second pillar pension provisions. The government is pulling back as a (fiscal) sponsor for these arrangements. It restricts the coverage of gross incomes up to 112,189 euros (for the year 2021, per annum), but pressures are mounting to lower this cap substantially.

Both the public and supplementary pension have been defined in terms of benefits, with premiums and taxes endogenously derived, whereas by 1 January 2023 a new pension system based on Defined Contribution (DC) plans pension system will replace the current defined benefit (DB) system, which is expected to give rise to more variable pension benefit outcomes than is currently the case. However, the new system also provides for some intergenerational risk sharing making this system de facto a collectively defined contribution (CDC) system.

### Governance

The governance of Dutch pensions is perhaps more complex than what is found abroad. The supplementary pension plans are agreed upon at a collective level in sectors of industry or within large companies between the so-called social partners: the employers or their representative organisation and the trade unions which represent the employees. Whenever an employer offers a supplementary pension scheme, participation by employees in that particular pension provider is mandatory. Although most Dutch employees accumulate their pension rights with pension funds, a small and increasing number of employees are covered by insurance companies offering DC pensions. Pension funds are non-profit organisations, where key policy decisions are made by the so-called social partners: the employer(s) or their representatives and the trade unions which represent the employees. Employees and pensioners of a pension fund can also be represented in the participants’ council, which gives solicited and unsolicited advice to the board of directors. However, in actual practice, most funds have outsourced their administration and/or asset management to for-profit pension organisations. To regulate the pension sector there are two organizations active to supervise pension funds and insurers: the central bank De Nederlandsche Bank (DNB) and the Dutch Authority for the Financial Markets (AFM). Under the Pensions Act and the Financial Supervision Act, the DNB closely monitors the financial and management operations of the pension providers. The task of the AFM is more limited but may gain more prominence in the new pension system. By law, pension providers are obliged to provide certain information to their stakeholders. The AFM checks that pension providers are meeting these requirements.

### Expectations

**Fig. 1:**
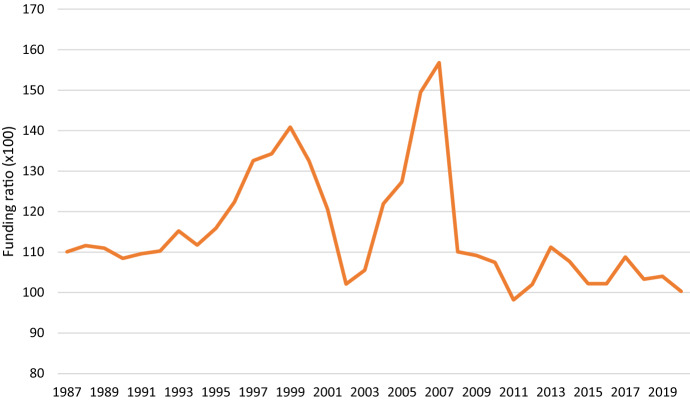
Average funding ratio of Dutch pension funds, 1987–2020. Note: The funding ratio is defined as the total assets divided by the total liability provisions made for (future) pensioners (‘voorziening pensioenvoorziening’) discounted by the risk-free market interest rate. *Source*: CBS Statline and DNB Pension statistics

In the Netherlands, approximately two thirds of the pension premium is paid or sponsored by the employer and the remaining third by the employee. By and large, most Dutch employees have until now a defined benefit pension plan. In the past, these benefits were promised in terms of a certain percentage (usually 70–75%) of an employee’s final gross pay based on 40 years of contributions. Over time, this ambition has been toned down to guaranteeing the benefit to a percentage of the average pay over the employee’s career. And during the last 10, 15 years, pension funds have come to realise that the promises they made in the past are now untenable and the indexation of pension rights and benefits has not been applied for the majority of pensioners. The funding ratio—the ratio of assets divided by the liabilities/promises—of pension funds captures the financial health of pension funds. It is however particularly vulnerable to developments in investments and interest rates. The increase in life expectancy, the various crises on the stock market, and the historically low interest rates, have made it difficult to match assets with future liabilities. The funding ratio is more or less designed to capture short or medium term capital market developments. However, when developments appear or turn out to be long-term, like zero or even negative interest rates, pension funds may find it difficult to take corrective action and only a lenient pension regulator can soften these worries. The development over time of the funding ratio of Dutch pension funds—total assets divided by the total liability provisions made for (future) pensioners—is presented in Fig. 1.

This figure clearly shows the impact of a number of crises on the financial sustainability of pension funds. The twenty-first century has been a volatile period for pension funds, which is a result of both the credit crisis and the subsequent crash on the stock market as well as the fall of interest rates to historically low levels. To interpret Fig. 1, one should keep in mind that a funding ratio of 100% implies that pension funds have no resources to index the pension rights of participants for inflation. Under the current rules of the pension law with a funding ratio of approximately 104 or lower pension funds should take corrective action in accordance with the Dutch pension regulator to bring the funding ratio to a safer level (in most cases between 104 and 110). And pension funds are allowed to index for inflation once the funding ratio has reached the level of 110 or higher. An earlier study in 2008 among trustees of Dutch pension funds Van Dalen et al. (2012) show that conservatism among board members may be at play in granting extra indexation of pension benefits. Their study shows that there are strong asymmetries in making decisions on indexation (and other key instruments). Even at a funding ratio of 130, which trustees at that time considered optimal, no or limited indexation was their preferred choice. Based on the strict regulation of the pension regulator DNB, one could say that the funding ratio is more or less internalized by the Dutch pension industry. A major reason why the pension regulator puts so much emphasis on strictly keeping an eye on the funding ratio is tied to the dominance of defined benefit pensions in the Dutch case which were seen for guarantees cast in stone. In order to keep those promises alive pension funds have to accumulate more buffers than those pension providers, like insurance companies, who offer defined contribution contracts. This aspect is perhaps illustrative of a country whose pension history is firmly based in defined benefit contracts which in turn lead to all kinds of dilemmas that are related how one views or perceives the pension contract, either as a complete or incomplete (or implicit) contract [cf. Clark and Monk (2008)].

Pension funds are required to maintain buffers that are capable of absorbing financial shocks. For example, if share prices fall sharply, such a buffer may prevent a pension fund from facing a funding deficit. The level of that buffer is expressed as the "required funding ratio". Pension funds are financially healthy if they meet the required funding ratio. The level of the required funding ratio is not the same for all pension funds. Those that take greater risks when making their investments have a higher required funding ratio, as they need a higher buffer to absorb financial setbacks. The level of the required funding ratio therefore reflects the risk level that a pension fund faces. In other words, this could mean that a pension fund may be perfectly healthy if its funding ratio is 110% or higher, whereas another is only financially sound at 120%.

The other central actor that we examine in this paper concerns the government as a key pension provider, which is relevant to understand trust certainly in the era which we are going to examine. The pension system was, ever since the start of the twenty-first century. under scrutiny, leading to a closing down of early retirement arrangements in 2005, but in the aftermath of the credit crisis of 2008, the Dutch government wanted to take concrete steps to think about reforming the pension system and in 2011 the government together with social partners agreed on increasing the statutory retirement age to 66 by 2020 and to 67 by 2025. One year later the plans were revised and the pension law was amended whereby the retirement age of 66 would now be achieved by the year 2019 and the age of 67 by the year 2023. However, in June 2015 the retirement age was increased even further. The government was encountering fiscal pressures, starting in 2012, from the Structural Growth Pact of the EMU as government debt level exceeded the threshold level of 60% and the long-run budgetary position did not look promising. Earlier research on acceptance of Dutch statutory retirement age reform by Parlevliet (2017) showed that up to 2012 the Dutch public supported a higher retirement age, but in 2013 this support plummeted. Increasing the public pension age was key is solving this pressure, and by the year 2022 the pension age would be linked to the average life expectancy at age 65. This abrupt change was met with a strong reaction by the unions. Especially older workers were caught by surprise (Van Solinge & Henkens, 2017), but the rapid increase in retirement ages also led to considerable concerns among employers (Van Dalen et al., 2019).

**Fig. 2:**
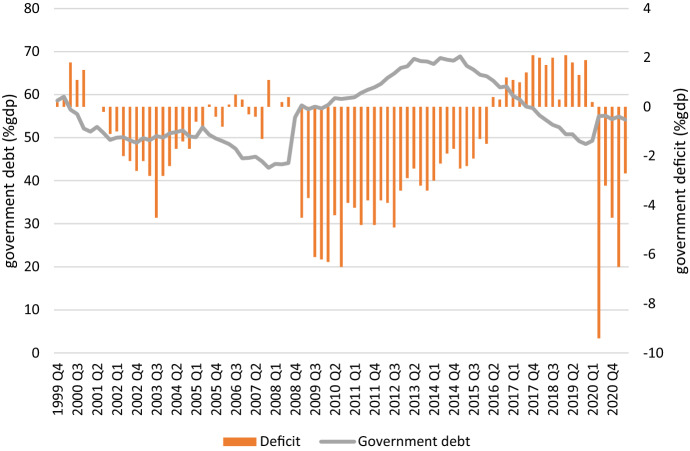
Government debt and budget deficits and surpluses in the Netherlands (% GDP), 1999 Q4-2021 Q2. *Source*: CBS Statline

As one can see in Fig. 2, the financial crisis led to an abrupt jump in the government debt ratio as well has years of relatively high budget deficits. By mid-year 2016 a series of budget surpluses were to be followed again by a steep rise of the government debt and a series of budget deficits to finance the consequences of the Covid crisis. In June 2019 the government and social partners finally agreed upon the transition to a new pension system. The new pension rules are expected to come into force no later than on 1 January 2023. Before 1 January 2027 at the latest, employers, employees and pension providers must have brought their pension schemes with pension build-up in line with the new system. Between 2023 and 2027, the employers’ organizations and trade unions and pension providers can make agreements about the new pension schemes and about the question on how to make the transition from the current Defined Benefit system to the new Defined Contribution system.

## Data and Methodology

### Data

We used data collected by surveys that were designed to measure trust in pension fund providers at eight points in time, to wit the years 2004, 2006, 2009, 2011, 2014, 2015, 2020 and 2021. Response rates of the various survey years vary from 65 to 81 percent (see “Appendix”). The fieldwork was carried out by the Centerdata of Tilburg University, initially through the Center-panel (2004–2015) and the for last 2 years through the LISS panel. Both are (primarily) internet panels. The number or respondents used by Center-panel surveys varies between approximately 1900–2100 respondents, whereas the LISS panel has in principle approximately 7500 individuals who can participate, although most sample sizes were tailor-made to specific projects as for the current project for the waves 2020 (1625) and 2021 (2876). All individuals in these panels were selected on the basis of a true probability sample of households drawn by Centerdata from the population register by Statistics Netherlands. The total sample in this study covers 16,352 respondents. The attrition rates in the panels are quite high so that these this dataset is not suitable for analyses of changes in trust over time at the individual level. In this article the data are analysed as a repeated cross-sectional survey dataset.

### Dependent Variables

Our central measures of trust concern the question whether respondents trust the two most dominant institutions in the Dutch pension system (1) pension funds as the (most dominant) organizations providing a supplementary pension; and (2) the government as the provider of a public pension (AOW). The questions that operationalize and capture trust in these institutions—pension funds and government—is the question: “To what extent do you trust the following institutions in offering a comfortable pension?”, with answer categories: (1) no trust; (2) little trust; (3) neutral; (4) some trust; and (5) a lot of trust. Distrust is defined as the state where respondents express little or no trust (1–2), and trust is the state where they express some or a lot of trust (4–5).^2^ Obviously these are broad-scope trust measures of pension funds and not trust measures of the specific pension funds of participants (Van der Cruijsen et al., 2021a). Because the general population is asked to respond to these questions, for some the trust question about pension funds will be somewhat abstract as they have no employment history and have thereby not accumulated supplementary pension rights., whereas the government as pension provider will by its very nature as provider of a public pension be more concrete as everyone is covered by a public pension.

To explain the development of trust over time we made use of the following set of variables: (1) year of birth, converted into specific birth cohorts^3^; (2) gender; (3) partner status (with partner or not); (4) highest attained educational level; (5) primary position on the labor market (employee, self-employed, disabled, unemployed, retired, and a residual group with other positions outside the official labor market (like student, those taking care of the household, volunteer, non-paid labor within the household or family business unpaid work with a social security benefit). The funding ratio in the quarter preceding the survey data collection period was used to capture the financial health of the pension funds. Where quarterly data were unavailable (at the start of the observation period), yearly data preceding the data collection period were used (see Table 9 in the “Appendix”). Table 1 provides an overview of the descriptive statistics of the variables used. This table shows that in the full sample, the level of trust in pension funds (47%) is higher than the trust in the government as pension provider (38%). Distrust is lower for pension funds (22%) than for the government as pension provider (30%).

**Table 1:** Descriptive statistics

	Frequencies (%)
Trust in pension funds as pension provider	
Distrust	22.3
Neutral	31.0
Trust	46.7
Trust in government as pension provider	
Distrust	29.8
Neutral	32.1
Trust	38.1
Year	
2004	12.6
2006	11.1
2009	12.4
2011	13.0
2014	13.1
2015	11.6
2020	9.6
2021	16.7
Age (in years)	Mean = 52.8 years (s.d. = 16.3)
Birth year^a^	
1920–1929	1.9
1930–1939	9.6
1940–1949	19.6
1950–1959	22.3
1960–1969	17.3
1970–1979	16.5
1980–1989	8.8
1990–1999	4.0
Labour force position	
Employee	46.5
Self employed	4.5
Pensioners	25.1
Unemployed	2.4
Disabled	4.3
Other	17.2
Level of education^b^	
Elementary	5.2
Lower vocational	24.4
Intermediate vocational	20.4
Intermediate general	11.2
Higher vocational	25.9
University	12.8
Gender	
Male (reference)	51.9
Female	48.1
Partner status	
No partner (reference)	24.9
Partner	75.1
N =	16,352

^a^See Table 7 for how the cohorts are distributed across the several survey years

^b^Educational categories are based on highest attained educational level: elementary = primary school; lower vocational = vmbo; intermediate general = havo, vwo; intermediate vocational = mbo; higher vocational = hbo; university

### Method

To be able to analyse trust and distrust as a discontinuous outcome variable we use multinomial logistic analyses in which the category ‘trust’ and the category ‘distrust’ are compared to the category of respondents who took a ‘neutral’ position.^4^ In analysing repeated cross-sectional survey data one should, first of all, be careful to *not* interpret the estimation results as giving insight into how *specific individuals* change their level of trust or distrust over the sample period 2004–2021. The present data structure gives primarily an insight into how the opinion of an aggregate population or group changes and how trust or distrust is affected by the financial stability of pension providers.

For analysing repeated cross-sectional surveys, we assume that an observation *y*_*i*_ (*i* = 1, …*I*)—the trust level in pension providers—for the respondent who is of age *j*, for the survey year of *t* obtained from the repeated cross-sectional survey, is specified using the following model:

1 $${y}_{i}=\alpha +{\varvec{\beta}}^{{\prime}}{{\varvec{X}}}_{i}+{A}_{j}+{P}_{t}+{C}_{n}+ {\varepsilon }_{i}$$

with *j* = 1,2, … *J*; *t* = 1, 2, …. *T*; *n* = 1,2, …. *N*; and where $\alpha$ is the constant term, **X**_i_ is the row vector representing all the relevant explanatory variables associated with observation i and ${\varvec{\beta}}\boldsymbol{^{\prime}}$ is the transpose of the row vector, *A*_j_ the effect of the *j*th year-old person; *P*_*t*_, the effect of the survey year *t*; *C*_*n*_ the effect of the *n*-th birth cohort; and ${\varepsilon }_{i}$ the disturbance term with mean zero and variance $\sigma$^2^. In the present case there are only three outcomes (distrust, neutral and trust) which are explained in a first step by gender, highest level of education, labour market position, partner status.

Using age-period-cohort (APC) analysis with repeated cross-sectional data involves some methodological challenges, as noted by a diverse set of authors, starting with Heckman and Robb (1985) and more recently by the work of Fosse et al. (Fosse & Winship, 2019; Fosse et al., 2020) and Bell and Jones (2014). The main issue revolves around the identification problem. This arises because there is an exact linear dependence between age, period and cohort (Period = Age + Cohort). Every solution to this problem gives the reader a second-best or third-best view of what is happening to some outcome variable in terms of these APC variables. Only by imposing strong assumptions can one solve this technical conundrum. As Bell (2020) notes in in a review article about the array of ‘solutions’ to the APC problem: “None of these methods solve the identification problem—rather they acknowledge that methods are limited by assumptions.” Heckman and Robb (1985) propose in dealing with this problem to assume specific measured variables that proxy the underlying unobserved variables. They assume the age or period or cohort effect is proportional to some other substantive variable.

In the present paper we restrict in our analysis the age parameters to zero and focus on the cohort effects in the realization that the estimates can reflect both age and cohort effects. And because we are mainly interested in discovering the issue of whether trust is related to the financial health of pension funds or the government, we will replace the year effects by proxy variables: average funding ratio in the case of pension funds and the government debt/GDP ratio in the case of the government. For both our models of trust/distrust in pension funds and that in government two versions are estimated. The first version includes the survey year as a predictor variable as well as a set of control variables that includes birth cohorts, the labor force position, the level of education, gender and partner status [cf. Parlevliet (2017)]. To correct for within respondent effects (i.e., the presence of respondents being in more than one wave^5^) we will use the clustering option to generate robust standard errors. In the second version we replace the year dummy variables by an indicator of financial stability: the funding ratio for the case of pension funds and government debt for trust in the government. In this second version we use robust standard errors allowing not only for within respondent correlation in the analyses but also for intra-year correlation by means of two-way clustering, to take care of the multilevel structure of the data.

## Results

The results of the multinomial logit analyses of trust and distrust in pension funds and the government are presented in Table 2 by means of average marginal effects, in other words the marginal effect of changing the values of covariates on the probability of observing a specific outcome (being distrustful, neutral or trustful). For instance, those having a university education decreases the average probability of being distrustful and neutral towards pension funds by 0.09, resp. 0.15 and it increases the average probability of having trust in pension funds by 0.24 (summing up by definition to zero), compared to the reference category of those with only elementary education.

**Table 2:** Explaining trust and distrust in pension providers by Dutch citizens in the years 2004–2021, average marginal effects^a^

	Trust in pension funds						Trust in government					
	Distrust		Neutral		Trust		Distrust		Neutral		Trust	
	Dy/dx	SE	Dy/dx	SE	Dy/dx	SE	Dy/dx	SE	Dy/dx	SE	Dy/dx	SE
Year (ref = 2004)												
2006	− 0.03**	0.01	0.00	0.01	0.04	0.01	− 0.03*	0.01	0.02	0.01	0.01	0.01
2009	0.07***	0.01	0.06***	0.01	− 0.13***	0.02	− 0,07***	0.01	0.02	0.01	0.05*	0.02
2011	0.06***	0.01	0.03*	0.01	− 0.09***	0.01	− 0.05***	0.01	− 0.01	0.01	0.06***	0.01
2014	0.09***	0.01	− 0.03*	0.01	− 0.06***	0.02	0.01	0.01	− 0.05***	0.01	0.04**	0.01
2015	0.12***	0.01	0.01	0.01	− 0.13***	0.02	0.10***	0.02	− 0.02	0.01	− 0.07***	0.01
2020	0.12***	0.01	− 0.01	0.01	− 0.10***	0.02	0.07***	0.02	− 0.06***	0.02	− 0.01	0.02
2021	0.06***	0.01	0.03*	0.02	− 0.09***	0.01	− 0.02	0.01	− 0.01	0.02	0.03*	0.01
Birth cohort (ref = 1920–1929)												
1930–1939	0.05	0.02	0.06*	0.03	− 0.11**	0.04	0.08**	0.03	0.07*	0.03	− 0.16***	0.04
1940–1949	0.08**	0.02	0.03	0.03	− 0.12**	0.04	0.14***	0.03	0.07*	0.03	− 0.21***	0.04
1950–1959	0.09***	0.02	0.04	0.03	− 0.13***	0.04	0.16***	0.03	0.07*	0.03	− 0.23***	0.04
1960–1969	0.14***	0.02	0.10**	0.03	− 0.24***	0.04	0.14***	0.03	0.10**	0.03	− 0.24***	0.04
1970–1979	0.17***	0.02	0.10**	0.03	− 0.28***	0.04	0.14***	0.03	0.09**	0.03	− 0.23***	0.04
1980–1989	0.18***	0.02	0.14***	0.03	− 0.32***	0.04	0.14***	0.03	0.12***	0.03	− 0.26***	0.04
1990–1999	0.14***	0.02	0.22***	0.04	− 0.36***	0.04	0.12**	0.03	0.17***	0.04	− 0.29***	0.04
Labour force (ref = employee)												
Self employed	0.12***	0.02	− 0.00	0.02	− 0.11***	0.02	0.05*	0.02	0.02	0.02	− 0.07***	0.02
Pensioners	− 0.04**	0.01	− 0.02	0.02	0.06***	0.02	0.02	0.02	− 0.01	0.01	− 0.01	0.02
Unemployed	0.04	0.02	0.02	0.02	− 0.06*	0.03	0.06*	0.02	0.03	0.02	− 0.08**	0.02
Disabled workers	0.06**	0.02	0.05*	0.02	− 0.11***	0.02	0.09***	0.02	− 0.03	0.02	− 0.06*	0.02
Other	0.01	0.01	0.03*	0.01	− 0.04**	0.01	0.01	0.01	0.01	0.01	− 0.02	0.01
Education (ref = elementary)												
Lower vocational	0.02	0.02	− 0.07**	0.02	0.05*	0.02	− 0.02	0.02	− 0.02	0.02	0.04	0.02
Intermediate vocational	− 0.02	0.02	− 0.08***	0.02	0.10***	0.02	− 0.05*	0.02	− 0.02	0.02	0.07***	0.02
Intermediate general	− 0.03	0.02	− 0.12***	0.02	0.15***	0.02	− 0.10***	0.02	− 0.05*	0.02	0.15***	0.02
Higher vocational	− 0.06***	0.02	− 0.12***	0.02	0.18***	0.02	− 0.12***	0.02	− 0.05*	0.02	0.17***	0.02
University	− 0.09***	0.02	− 0.15***	0.02	0.23***	0.02	− 0.17***	0.02	− 0.10***	0.02	0.26***	0.02
Gender (ref = male)												
Female	0.01	0.01	0.06***	0.01	− 0.07***	0.01	− 0.02	0.01	0.06***	0.01	− 0.04***	0.01
Partner (ref = none)												
Partner	0.01	0.01	− 0.00	0.01	− 0.01	0.01	0.04***	0.01	− 0.02*	0.01	− 0.02*	0.01
Pseudo R^2^	0.05							0.03				

N = 16,352. Estimated with multinomial logit with neutral category as the reference category.; ***p < 0.001; **p < .01; *p < 0.05. Dy/dx = average marginal effects of covariates (x) on outcomes of distrust, neutral, and trust (y)

^a^Standard errors controlled for cluster effects at respondent level. Due to rounding errors the marginal effects across outcomes may not some up to zero

**Fig. 3:**
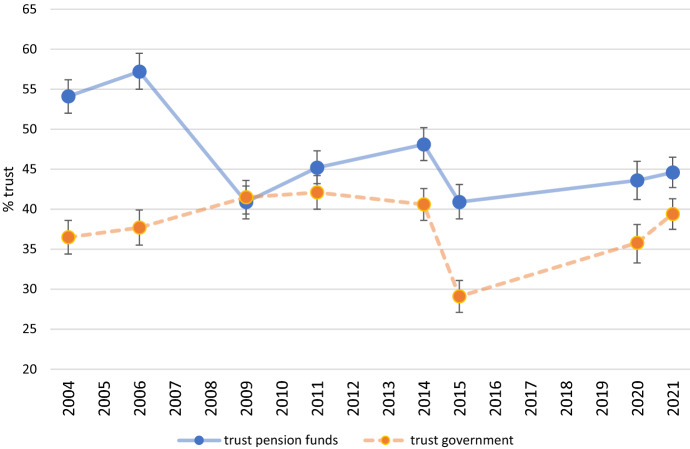
*Trust* in pension funds and government across time. *Note*: Trust levels are predicted margins based on models presented in Table 2. Bars denote 95% confidence intervals

**Fig. 4:**
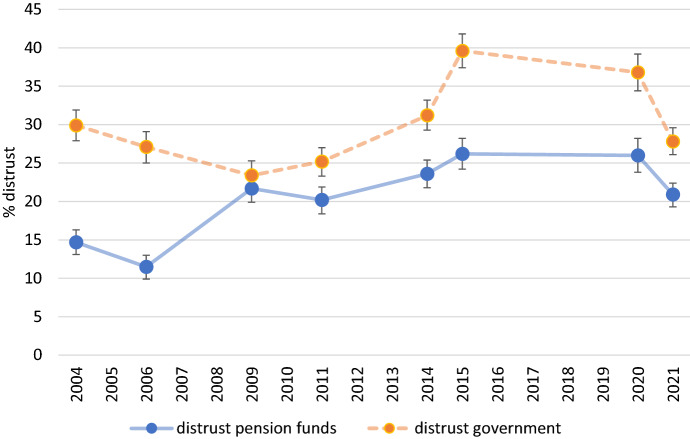
*Distrust* in pension funds and government across time. *Note*: Trust levels are predicted margins based on models presented in Table 2. Bars denote 95% confidence intervals

The first three columns contain the results for the case of pension funds and the fourth to sixth columns contain results the corresponding models for the case of the government in its role as provider of the public pension. The coefficients in both models show that trust and distrust in pension funds as well the government differ over time. To get a more refined insight of this development the predicted margins for trust levels across years (controlling for all individual level variables included in the model) have been presented in two figures. Figure 3 displays the percentage of trust in pension funds and the government for subsequent survey years between 2004 and 2021. Figure 4 displays the distrust levels in pension funds and the government across the same sample period.

As one can see in these figures, at the start of the twenty-first century citizens had considerable trust in pension funds and were considerably distrustful about the government as pension provider. With the emergence of the credit crisis in 2008, this level of distrust about the government became less prominent and *trust* in pension funds *steeply declined* and *distrust increased considerably*, but in the years after the crisis people regained some trust in pension providers, although the distrust did not decline. It is, however, noteworthy to see that in times of crisis the trust in these two institutions can switch rank: whereas pension funds experienced a steep decline in trust, the government gained some trust. In 2009 both the government and pension funds were considered to be equally trustworthy in the eyes of citizens. This is a unique moment in time because in normal, non-crisis times the government has a significant *lower* level of trust compared to pension funds. This ‘crisis effect’ on the level of trust in government could be due to the fact that the Dutch government in 2009 came to the rescue of banks that were ‘too-big to fail’ (like ING and ABN AMRO) and indirectly prevented a further crash for pension funds.

The sudden drop in trust in both pension funds and the government between 2014 and 2015 can be ascribed to the fact that the Dutch government agreed to speed up the rate at which statutory retirement ages were set to increase, thereby lowering the long-term government expenditures on public pensions.^6^ Because supplementary pensions and the public pension are intertwined, and the blame for increasing the public pension age seems to have been shifted to the government and to a far lesser extent to the pension funds. Figures 3 and 4 show a widening of the gap in trust and distrust between these two pension providers. After 2015 we see a slight recovery in trust in the government which might be related to the adjusted pension reform concluded in 2020 in which the government reduced the pace of the increase of the public pension age in response to political pressure from various sides.

Next to the year dummies in Table 2, several individual level factors proved to be relevant to understand how trust in pension providers is differently perceived by citizens. The results in the first column shows that distrust in pension funds is more likely and trust more unlikely among younger birth cohorts, self-employed and those who are disabled. The result that young birth cohorts are being more distrustful than older cohorts may be related to the fact that their involvement and interest in pensions is often low (see Hershey et al., 2017 and Van Raaij et al., 2011), but it can also be a reflection of the fact that the demographic pressure of old aged relative to the working age population has become higher over time. Certainly for the case of public pension which are financed on a pay-as-you-go basis this grey pressure ratio may be a reason for becoming more distrustful to pension promises. The result that self-employed are particularly distrustful is a novel element which might be a result of the fact that self-employed are often excluded from participating in pension funds once they change their status from employee to self-employed. And in case they want to accumulate pension reserves they are confronted with the fact that the pension premium is far higher than the privileged position of employee (in the Netherlands on average the employer pays two thirds of the total premium and the employee one third). But it may also signal a characteristic of the self-employed who are sometimes forced into this position because of reorganizations within firms or downsizing (Hershey et al., 2017) or because some self-employed are not truly entrepreneurs: according to Kwon and Sohn (2021) self-employed and entrepreneurs tend to work in different trust settings, with the self-employed in settings that are highly monitored and they meet what Rousseau et al. (1998) calls ‘calculus-based trust'; a form of trust based on rational choice, focused on the short run (contrary to relational trust, with repeated interaction).

The results in the third column of Table 2 show that these structural indicators are more important in understanding the differences in *trust* in pension among different members in society. Younger birth cohorts are compared to older birth cohorts less likely to be in the trust category.^7^ Furthermore, disabled workers and self-employed are less likely to display trust in pension funds. The educational gradient is strong, with in particular higher educational levels being much more likely to have trust in pension funds. Table 2 also reveals that women are more likely to belong to the neutral category; they are less likely to trust pension funds. This gender difference may be partially a reflection of different interests in or knowledge of pension issues among men and women in the Netherlands (Van Raaij et al., 2011). For a long time, the labor market histories of women differed considerably and perhaps they still do as part-time work is still a prominent characteristic of Dutch labor force participation of women.^8^

The results of trust and distrust in pension provision by the government are presented in the fourth to sixth column of Table 2. These results show that distrust is more likely among disabled workers and less likely among the higher educated. Also, with respect to the pension provision of the government, self-employed are less likely to express trust and women, just as in the case of pension funds, are more likely to take a neutral position.

In Table 3 the results are presented of the models in which the year dummies (of Table 2) are replaced by indicators of financial stability as a proxy for underlying unobserved variables. For the multinomial logit model on trust/distrust in pension providers we included the average funding ratio of Dutch pension funds as a group as predictor. And in the corresponding model for the case of (public) pension provision by the government we included government debt (as a percentage of GDP) as a predictor variable.

**Table 3:** Explaining trust and distrust in pension providers by Dutch citizens in the years 2004–2021 with proxy variables for financial stability, average marginal effects^a^

	Trust in pension funds						Trust in government					
	Distrust		Neutral		Trust		Distrust		Neutral		Trust	
	Dy/dx	SE	Dy/dx	SE	Dy/dx	SE	Dy/dx	SE	Dy/dx	SE	Dy/dx	SE
Funding ratio (× 10^–2^)	− 0.27	0.16	− 0.17	0.12	0.44***	0.06	–	–	–	–	–	–
Government debt ratio (% gdp) (× 10^–2^	–	–	–	–	–		0.24	0.26	− 0.15	0.13	− 0.08	0.26
Birth cohort (ref = 1920–1929)												
1930–1939	0.05*	0.02	0.07	0.05	− 0.12*	0.05	0.08*	0.03	0.08	0.04	− 0.16**	0.06
1940–1949	0.10***	0.02	0.04	0.04	− 0.14**	0.04	0.15***	0.03	0.07*	0.03	− 0.22***	0.04
1950–1959	0.12***	0.02	0.04	0.03	− 0.16***	0.04	0.18***	0.03	0.06*	0.04	− 0.24***	0.05
1960–1969	0.17***	0.03	0.10**	0.04	− 0.27***	0.04	0.16***	0.04	0.09*	0.04	− 0.25***	0.04
1970–1979	0.20***	0.03	0.10**	0.03	− 0.30***	0.03	0.15***	0.03	0.08*	0.04	− 0.23***	0.04
1980–1989	0.22***	0.03	0.13***	0.03	− 0.35***	0.03	0.17**	0.05	0.10*	0.04	− 0.27***	0.05
1990–1999	0.18***	0.03	0.21***	0.04	− 0.39***	0.05	0.14***	0.04	0.14**	0.05	− 0.29***	0.05
Labour force (ref = employee)												
Self employed	0.12***	0.02	− 0.00	0.02	− 0.12***	0.02	0.05***	0.01	0.02	0.02	− 0.07**	0.02
Pensioners	− 0.01	0.02	− 0.02	0.01	0.04	0.03	0.04	0.03	− 0.02	0.02	− 0.02	0.02
Unemployed	0.05*	0.02	0.01	0.02	− 0.07**	0.02	0.07**	0.02	0.02	0.01	− 0.09***	0.03
Disabled workers	0.07***	0.02	0.05**	0.02	− 0.12***	0.01	0.09**	0.03	− 0.03	0.02	− 0.06*	0.03
Other	0.01	0.02	0.03**	0.01	− 0.04*	0.01	0.01	0.02	0.01	0.02	− 0.02	0.021
Education (ref = elementary)												
Lower vocational	0.03	0.02	− 0.07***	0.02	0.04*	0.02	− 0.01	0.02	− 0.02	0.02	0.04*	0.02
Intermediate vocational	− 0.01	0.02	− 0.09***	0.02	0.09***	0.03	− 0.04**	0.01	− 0.03	0.01	0.07***	0.01
Intermediate general	− 0.03	0.02	− 0.12***	0.02	0.14***	0.02	− 0.10***	0.02	− 0.05*	0.02	0.15***	0.02
Higher vocational	− 0.05*	0.03	− 0.12***	0.02	0.18***	0.03	− 0.12***	0.02	− 0.05**	0.02	0.17***	0.02
University	− 0.08**	0.02	− 0.15***	0.02	0.23***	0.02	− 0.17***	0.02	− 0.10***	0.03	0.27***	0.03
Gender (ref = male)												
Female	0.01	0.01	0.06***	0.01	− 0.08***	0.01	− 0.01	0.01	0.06***	0.01	− 0.04***	0.01
Partner (ref = none)												
Partner	0.01	0.01	− 0.00	0.01	− 0.01	0.01	0.04**	0.01	− 0.02	0.01	− 0.02	0.01
Pseudo R^2^	0.04		0.02									

N = 16,352. Estimated with multinomial logit with neutral category as the reference category; ***p < 0.001; **p < .01; *p < 0.05. Dy/dx = average marginal effects of covariates (x) on outcomes of distrust, neutral, and trust (y)

^a^Standard errors controlled for cluster effects within years and at respondent level, by means of two-way clustering. Due to rounding errors the marginal effects across outcomes may not some up to zero

The results show that the average marginal effect of a high funding ratio is associated with more people having trust in pension funds and at the same time less people who are distrustful or neutral.^9^^,^^10^ The average marginal effect of the level of the funding ratio on being distrustful (and neutral) are not statistically significant, which suggests that one can predict what happens with trust as the funding ratio changes but where this increase or decrease exactly comes from—distrust or neutral—is not clear. Of course, respondents may show different reactions because they are not all firmly tied to a pension fund compared to the employees who are by default tied to a pension fund. To check whether respondents with different labor market positions react differently with respect to the funding ratio we also have run interactions between the funding ratio and the six labor market statuses. The outcome of this analysis is that only pensioners show a slightly higher response to variability in funding ratios (at *p* = 0.02). The other labor market positions do not show differences in reaction compared to those of employees. This higher sensitivity to funding ratios of pensioners makes seems plausible because pensioners are often more interested in what happens with their pension income and they are, contrary to those of working age, in a position to see on their banking account what their pension benefit really amounts to.

The fact that the funding ratio matters is not to be interpreted as an indication the Dutch understand and know the ins and outs of the funding ratio, but more that it has signal function and that there may be thresholds which have financial consequences for their pension rights. To test for an alternative indicator that captures this signaling aspect, we used two alternative proxies: (1) the percentage of all pension funds in the Netherlands in the danger zone (funding ratio < 104) as registered by DNB with a funding ratio lower than 104; and (2) for the specific years the funding ratios are divided into three classes to reflect some kind of non-linearity in regulatory policies: (1) lower than 104; (2) 104–110; and (3) more than 110. These tests lead one to conclude that the higher the percentage of funds in the danger zone the less likely respondents will express trust (at *p* < 0.01) and the more likely one distrust pension funds in general (see Table 4 with only the new proxy variable). The second proxy variable shows that when the funding ratio clearly is in the healthy zone (> 110) one can see that trust increases mainly coming from a decrease of those respondents who were neutral.

**Table 4:** Explaining trust and distrust in pension funds by Dutch citizens in the years 2004–2021 with alternative proxy variables, average marginal effects

	Trust in pension funds					
	Distrust		Neutral		Trust	
	Dy/dx	SE	Dy/dx	SE	Dy/dx	SE
Variant 1						
% Pension funds in danger zone^a^	0.11**	0.04	0.04	0.03	− 0.15***	0.04
Variant 2						
Funding ratio < 104 = ref						
104–110	0.01	0.03	− 0.04**	0.01	0.03	0.03
> 110	− 0.03	0.04	− 0.06***	0.01	0.09**	0.03

***p < 0.001; **p < .01; *p < 0.05

^a^Percentage of all pension funds in the Netherlands with a funding ratio of 104 or lower (source DNB). Same model as Table 3 with replacement of funding ratio by % funds in danger zone and same set of covariates

When we turn to the effect of government debt levels on trust/distrust in government, the results show that a higher or lower government/debt ratio is not associated with having distrust or trust in the government as a pension provider. Of course, government debt is—compared to the funding ratio of pension funds—an imperfect measure as the accumulated government debt is the result of various government outlays not just the public pension expenditures. We also experimented by using government budget deficits (as a percentage of GDP) as an alternative predictor but this also did not affect the level of trust and distrust in the government as a pension provider.

**Fig. 5:**
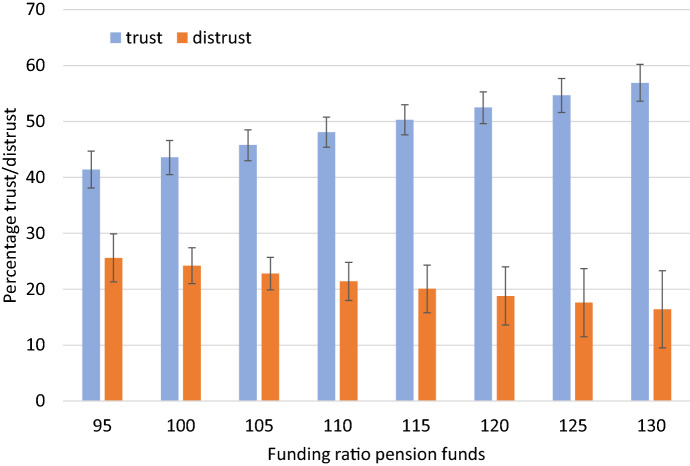
Levels of trust and distrust in pension funds for various funding ratios. *Note*: Trust levels are predicted margins based on models presented in Table 3. Interval bars denote 95% confidence intervals

To capture the size of the effects of the estimated response of trust to changes in the average funding ratio of pension funds in more detail we present Fig. 5: the predicted margins of trust and distrust (based on the model in Table 3) related by the funding ratio of pension funds (ranging from 95 to 130).

The figure shows that the level of trust is clearly associated with the average funding ratio of pension funds. With a funding ratio of 95, the percentage of respondents showing trust in pension funds is predicted to be 41%. With a funding ratio of 130, the percentage of respondents showing trust is predicted to be 57%. Achieving a situation where the majority of the adult population trusts pension funds is, based on the estimation results, attained at funding ratios of around 115 or higher.

The story is however slightly different for the case of distrust. Although we see a decrease in the percentage of distrust across the various funding ratios—from 26% in case of a low funding ratio of 95 to 16% in case of a high funding ratio of 130, these differences are smaller than in the case of trust and not statistically significant (see first column of Table 3). These findings suggest that some asymmetry in the relationship between the funding ratio and trust and distrust exists; a high funding ratio is associated with a high trust level, but a high funding ratio is not clearly associated with a low level of distrust as displayed in the figure by the wider confidence intervals for the low ender and the higher end of the depicted funding ratios.

## Pension Policy Implications

What are the lessons to be learned from comparing the trust people have in pension funds and the government? And what might be the implications for pension policy?

### Funding Ratios of Pension Funds Matter for Trust

We will start with the broad-based message that an indicator of financial health of pension funds—the funding ratio—*matters* in the eyes of the general population. Of course, it does not automatically make a pension fund trustworthy in the eyes of all, as some remain distrustful in good times and some trustful even in hard times. But regaining trust by improving the financial soundness of pension funds lies within the possibilities of pension funds.

### Government Finance doesn’t Matter for Trust in the Public Pension Provider

We also considered the issue of trust in the most basic provider of a pension, viz. the government. For the period under review estimates show that the indicator of financial soundness of the government—public debt as a percentage of GDP—was not associated with the level of trust or distrust that the population has in the government as pension provider. An alternative indicator—the fiscal deficit (% GDP)—also did not show a clear association. To some extent this is surprising compared to the indicator of pension funds, because both the funding ratio and the public debt level can have consequences once they exceed a threshold level. Our interpretation why we see this different reaction across pension providers may be because the funding ratio of pension funds below a certain threshold has direct consequences for the group of pension participants, whereas a public debt level exceeding the threshold of 60% of GDP has consequences that are dispersed across the population. Of course, once the consequences of exceeding this threshold are focused on the pension domain—as was the case in 2015 in speeding up the increase of the public pension age—trust is deeply affected. This goes to show that financial indicators may not tell the entire story and this is also the reason why during the COVID-19 crisis the soaring public debt level did not directly impact trust in the government as the Structural Growth Pact rules were not perceived as applicable to the exceptional circumstances of this crisis.

### Different Propensities to Trust Pension Institutions Among the Population

Pensioners generally have a higher level of trust than employees, self-employed are less trusting than employees, and the lower educated are also less trusting than the higher educated. And although one cannot pinpoint this exactly, there are signs that older generations are more trusting than younger generations. This difference could be a reflection of the transition that has been made in the Dutch pension system, but it could also be that for employees they still have to wait and see what becomes of their pension savings, whereas pensioners see what those savings actually amount to as they receive their pension benefits. These differences in propensities to trust make it difficult to communicate pension measures in particular for the government, but also for pension funds.

### Future Implications for Policy Reform

The transition to a new pension system in the Netherlands, a reform involving a shift from DB contracts to a DC contract with some form of intergenerational risk sharing or an (improved) DC contract (see Metselaar et al. (2022)) raises two issues that are related to trust:

1. Searching for new indicators of sound pension finance

Given that pension promises in the new situation are no longer explicitly guaranteed, the use of the funding ratio will fade away. Still our analyses show how people more or less have internalized the funding ratio as a measure of financial health of pension funds. The open question is which indicator or set of indicators or labels will become the new touchstone for judging whether a pension package is good and which is under par? It would seem that this could potentially alter the pension fund landscape radically as this will push an urge to competition among pension funds.

2. Trust and distrust matter for making individual choices

The new pension system will offer more options for choice. Participants will have more freedom to choose with respect to timing of pensions, an option to take-up a lump sum (at most 10%) of accumulated pension wealth and depending on the type of DC contract to accept more variability in pension income. While choice in the eyes of a marketing expert may be seen an unequivocally good, there are reasons to be prudent in offering choice as trust or distrust of participants may generate side-effect not envisioned by introducing them. For instance, Van der Cruijsen and Jonker (2019) show how workers and pensioners who do not trust their pension fund are more likely to prefer a lump sum over annuity-based arrangements. And to cite another example pointed out by Van Dalen et al. (2022), the new pension system will make pension benefits more uncertain. They show a clear association between the dislike of participants for uncertain outcomes and their intended take-up rate of the lump-sum option. And as a final implication, in the upcoming reform more options are provided for self-employed to participate on a voluntary basis in the pension fund of their particular sector of work. The lower level of trust of self-employed may be a sign that their interest in participating in pension funds will be modest.

## Conclusion and Discussion

Trust and distrust in pension providers in the Netherlands at the start of the twenty-first century has been shown to be volatile. Before the Great Recession the level of trust was high and the level of distrust low. Once the consequences of the crisis became clear for pension funds trust dropped considerably and distrust increased. Financial stability of pension funds as measured by the funding ratio -played a role in understanding the development of trust and distrust. For the case of government, financial stability—in terms of government debt/GDP ratio—does not appear to be linked to trust of the population. Our estimates suggest that the level of the funding ratio affects those who distrust and those who trust asymmetrically. This is an important conclusion as it *suggests* that once trust is lost and people become distrustful it will probably become harder to persuade them by simply increasing the funding ratio. This fits in with the results of a recent study by Van Dalen and Henkens (2021) showing that an estimated 15% of the Dutch population expresses low levels of trust in *all* political and societal institutions and this is strongly associated with a low level of broad-scope trust in pension funds. For the day-to-day practice of pension funds, this could mean that as distrust grows it might become increasingly difficult to win the hearts and minds of those who express low levels of trust in societal institutions in general.

### Limitations

Of course, the stated findings in this paper are bound by some of the limitations of the dataset (and the time period) used. First, the number of repeated cross-sectional surveys is limited to eight years covering a time span of 18 years and extending this study by an extra number of years would have generated perhaps more robust insights. Second, the associations between funding ratios and level of trust do not permit us to make claims about which transmission mechanisms are at play in generating trust at the micro-level. However, some other research gives a clue that the pension participants are likely to appreciate the consequences of having a high or a low funding ratio. For individual pension fund data we have shown earlier (Van Dalen & Henkens, 2015) that downgrading pension rights—a policy decision that has to be taken once the funding ratio becomes too low—clearly increases distrust and lowers trust in a specific pension fund. Van Zaal (2017) shows in a more refined manner for pension fund participants how indexation and downgrading of pension rights can respectively increase and decrease trust compared to those participants who do not experience a change in pension rights. However, one should be aware that besides the financial consequences there can be various reasons why citizens trust financial institutions (Van Dalen & Henkens, 2018; Van Esterik-Plasmeijer & Van Raaij, 2017) and future research has to await how this can be refined in the case of pension providers. And third, one should remember that this paper focusses on broad-scope trust in pension funds. This is in general lower than the trust employees and former employees have in their own pension fund (cf. Van der Cruijsen et al. (2021b)).

### Discussion

The relevance of the current empirical study in understanding the development of trust is of some concern as policy makers are tempted to think that increasing the funding ratios of pension funds will completely regain the trust that was present during the golden ages of pension funds, when funding ratios of 120 or higher were quite common (and at one time reaching even the level of 158). The Dutch pension reform that is proposed to take place in the coming years will put the trust in the pension system and its providers to the test.

One of the lessons one can derive from is that trust in pension funds is based on outcomes that are associated with outcomes on financial markets, outcomes that are condensed in indicators like the funding ratio. The trouble with this reliance is that, although to some a considerable degree financial risks are manageable for funds, there are also outcomes associated with crises that are not manageable. This is connected with the failure of financial organizations to recognize what John Maynard Keynes (1937) called “irreducible uncertainty”: uncertainty that cannot be reduced to statistical probabilities. Or put differently, there will always be “unknown unknowns.” Insurance companies and pension funds make decisions as if they do know the relevant risks, but this convention will only hold in normal times. As soon as a society is in a state of flux—when a war erupts, a state or a city is flooded, or a pandemic spread—anything can happen, and the rules and conventions are no longer of use. In extraordinary times, one can expect extraordinary policies, like Quantitative Easing as practiced by the ECB or the Fed, giving rise to zero or negative interest rates. In the Dutch context this puts funding ratios of pension funds under pressure hence creates the circumstances for a steady fall in trust.

Another lesson we may learn from the past decades is that trust is likely to fall when radical changes occur in a pension system. Especially for a system that has basically has remained the same for 50 years this reform has come as a shock and an upcoming fundamental reform still has to be carried out in the upcoming five years. Many fear that they will lose some pension rights and such a loss of pension rights will undoubtedly lead to a loss of trust. However, it may matter whether such a loss is caused by outside or natural forces, like population ageing or capital markets developments, or by inside or interpersonal forces, like pension reforms or regulatory policies. It is what Fehr (2009) calls betrayal aversion, which has far deeper and enduring consequences than the ‘natural’ causes. It is beyond the bounds of this paper to establish this phenomenon, but it is certainly a risk of a radical pension reform that has to be taken account of in the preparation of such a reform.

## Appendix

See Tables 5, 6, 7, 8 and 9 .

**Table 5:** Sampling properties

Sample	Timing fieldwork	N	Response rate
2004 (CentERpanel)	November	2070	65%
2006 (CentERpanel)	October	1823	69%
2009 (CentERpanel)	January	2039	73%
2011 (CentERpanel)	March	2129	79%
2014 (CentERpanel)	June	2145	71%
2015 (CentERpanel)	July	1934	71%
2020 (LISS panel)	February	1625	81%
2021 (LISS panel)	February–March	2876	81%

**Table 6:** Frequencies of trust levels in pension funds and the government (as pension providers), 2004–2021

	Periods								
	2004	2006	2009	2011	2014	2015	2020	2021	Total average
*Pension providers*									
Trust in pension funds									
No trust	2.9	3.7	5.3	3.4	4.2	5.8	5.4	4.6	4.4
Little trust	13.2	9.2	18.0	18.0	21.6	22.2	23.5	18.2	17.9
Neutral	29.1	29.3	35.1	31.8	26.4	30.2	29.8	34.4	31.0
Some trust	40.4	40.5	32.5	36.7	36.7	33.4	33.1	32.6	35.7
Lot of trust	14.4	17.3	9.1	10.1	11.0	8.5	8.1	10.2	11.1
	100.0	100.0	100.0	100.0	100.0	100.0	100.0	100.0	100.0
Trust in government									
No trust	6.9	8.2	5.1	5.8	6.0	12.6	9.4	7.4	7.5
Little trust	23.0	18.8	18.4	19.6	24.6	28.0	27.3	20.2	22.3
Neutral	33.0	34.6	34.7	32.0	28.0	30.9	28.6	34.0	32.1
Some trust	30.1	30.3	34.4	34.8	34.5	24.9	30.0	32.4	31.6
Lot of trust	7.0	8.1	7.4	7.9	6.9	3.6	4.7	6.0	6.5
	100.0	100.0	100.0	100.0	100.0	100.0	100.0	100.0	100.0

**Table 7:** Two-way cross-classified data structure in pension trust data—number of observations in each cohort-by-period cell

Birth cohorts	Periods								
	2004	2006	2009	2011	2014	2015	2020	2021	Total
1920–1929	92	66	55	44	24	17	6	10	314
1930–1939	285	252	254	253	190	185	62	92	1573
1940–1949	374	310	425	478	405	454	274	483	3203
1950–1959	468	420	443	517	398	466	332	605	3649
1960–1969	397	305	332	380	318	289	300	501	2822
1970–1979	310	335	362	320	493	301	204	375	2700
1980–1989	133	119	108	91	268	156	209	348	1432
1990–1999	0	6	48	40	48	33	176	308	659
Total	2059	1813	2027	2123	2144	1901	1563	2722	16,352

**Table 8:** Trust (% some/a lot of trust) in pension funds and government by birth cohorts, across sample years

Birth cohorts	2004	2006	2009	2011	2014	2015	2020	2021
Trust in government								
1920–1929	50%	68%	59%	–	–	–	–	–
1930–1939	49%	46%	51%	47%	34%	21%	43%	37%
1940–1949	37%	43%	41%	40%	37%	26%	26%	35%
1950–1959	30%	32%	40%	40%	38%	26%	32%	39%
1960–1969	33%	35%	33%	43%	40%	27%	34%	38%
1970–1979	34%	38%	43%	46%	40%	29%	38%	36%
1980–1989	31%	21%	31%	38%	37%	33%	34%	37%
1990–1999	–	–	–	–	–	–	40%	37%
Trust in pension funds								
1920–1929	67%	60%	65%	–	–	–	–	–
1930–1939	71%	72%	50%	54%	50%	44%	51%	46%
1940–1949	60%	68%	46%	50%	60%	47%	39%	49%
1950–1959	52%	55%	43%	44%	52%	44%	55%	54%
1960–1969	45%	51%	33%	39%	39%	31%	40%	39%
1970–1979	44%	46%	31%	43%	35%	31%	34%	34%
1980–1989	40%	34%	32%	30%	32%	35%	34%	32%
1990–1999	–	–	–	–	–	–	32%	27%

Cells with less than 50 observations are not reported. Weighted by age, gender and education

**Table 9:** Data used in measuring time effects in trust

Sample	Timing fieldwork	Government debt (% gdp)	Government deficit (% gdp)	Funding ratio pension funds
2004	November	51.7	− 1.4	105.5
2006	October	47.4	0.5	127.3
2009	January	54.7	0.0	95.5
2011	March	59.2	− 3.9	106.7
2014	June	67.1	− 3.0	111.2
2015	July	66.7	− 2.4	108.9
2020	February	48.5	1.9	104.0
2021	February–March	54.3	− 4.5	100.3

Based on the level registered in the quarter preceding the fieldwork

## Footnotes

1 In economic theory trust is always involved in economic interactions and is often captured in game theoretic terms like so-called trust games; games in which actions of trustors reveal their trust by investment decisions, which can be reciprocated or abused by the trustee [see Berg et al. (1995)]. See Johnson and Mislin (2011) for a meta-analysis of the various experimental outcomes.

2 For a full overview of the distribution of answers across the various sample for these five categories, see “Appendix” Table 6.

3 The number of observations per cohort across the various samples is presented in Table 7 in the “Appendix”, as well as the levels of trust across cohorts for the various sample years.

4 To test the proportional odds assumption underlying an ordered logistic analysis we have used the Brant-test (Brant 1990) which shows that this form of analysis violates the parallel regression assumption. The multinomial logit is in that respect a more appropriate form of regression analysis.

5 Of the total sample 38 percent of the respondents appears more than one time in the dataset.

6 The pension law (‘Wet verhoging AOW- en pensioenrichtleeftijd’) was approved by the First Chamber of Parliament on June 4, 2015 and the survey was held in July 2015. The surprise and dismay among workers (especially older workers close to retirement) was in that respect an element that may explain the sharp fall in trust as measured between June 2014 and July 2015. The initially announced stepwise increase in the public pension age was apparently not that disturbing, but the fact that government broke an earlier promise/plan may have led to this drop in trust (cf. De Beer et al., 2017).

7 These cohort effects are difficult to truly pinpoint to belonging to a cohort as noted in the methodology section on the APC identification problem. This can also be seen as an age effect as similar models using age group dummies instead of cohort dummies shows. The older respondents are, the more they trust pension providers.

8 Unfortunately we could not check this further for women working full-time, because we have no information on the number of hours of the employees in question.

9 As a robustness check for the funding ratio effect, we also estimated a version that includes age as a continuous variable, which yielded the following set of marginal effects of the funding ratio: distrust − 0.22 (s.e. 0.14); neutral − 0.19 (s.e. 0.10) and trust 0.41 (s.e. 0.09). Hence the funding ratio coefficient hardly changes, the age coefficient is insignificant for all outcomes (p < 0.05) and the cohort effects are also not affected in a significant manner.

10 Interaction terms between the variables for age cohort and financial indicators government/pension funds as well as the interactions for age cohort and year are jointly statistically insignificant.
